# Supplementary material for: Assessment of Luteal Function Using Rectal Palpation, B-Mode Ultrasonography, and Progesterone Determination to Improve Recipient Selection in Embryo Transfer Programs
Source: Animals (Basel). 2023 Sep 9;13(18):2865. doi: 10.3390/ani13182865 (PMC10525574; doi:10.3390/ani13182865)
Supplement: Supplementary file 1 [file animals-13-02865-s001.zip › animals-2514980-supplementary.pdf]

Supplementary Materials

Table S1: Embryo management, according to the cryoprotectant agent, to perform the embryo transfer in Holstein heifers.

Embryo preservation	Management description
**Fresh**	1. After collection, the embryos were washed according to the IETS recommendations<br>2. The embryos were placed into a sterile 0.25 mL straw as follows: cotton end, conservation medium column, air column, conservation medium column, air column, conservation medium with embryo column, air column, conservation medium column<br>3. Transfer to the recipient
**Ethylene glycol**	1. The straw was air – exposed for 6 seconds<br>2. It was introduced 30 seconds in water at 30ºC<br>3. Direct transfer
**Glycerol**	1. The straw was air – exposed for 6 seconds<br>2. It was introduced 30 seconds in water at 30ºC<br>3. The content of the straw was placed on a Petry dish and a thawing 4 – steps procedure was followed (EMCARE™, ICP$_{bio}$, Auckland, New Zealand)<br>4. First step: the embryo was placed in a medium containing 6 % glycerol and 10.3 % sucrose for 5 minutes<br>5. Second step: the embryo was placed in a medium containing 3 % glycerol and 10.3 % sucrose for 5 minutes<br>6. Third step: the embryo was placed in a medium containing 10.3 % sucrose for 5 minutes<br>7. Fourth step: the embryo was placed in a maintenance medium for 5 minutes<br>8. The embryo was placed into a sterile 0.25 mL straw for its subsequent transfer
